# Analysis of growth variations and expression patterns of auxin response factor gene family in *Fallopia multiflora* under different light qualities

**DOI:** 10.3389/fpls.2025.1645778

**Published:** 2025-12-10

**Authors:** Wenze Liu, Shengwei Zhou, Yuxin Zheng, Wenchuan Hou, Leixia Chu, Ning Dong, Ruihang Zhang, Mengping Wang, Xupeng Gu, Jie Wan, Shouhua Liu, Linlin Yang, Chengming Dong

**Affiliations:** 1 Henan Provincial Ecological Planting Engineering Technology Research Center of Daodi Herbs, School of Pharmacy, Henan University of Chinese Medicine, Zhengzhou, Henan, China; 2 Henan University of Chinese Medicine, The Engineering and Technology Center for Chinese Medicine Development of Henan, Zhengzhou, Henan, China; 3 Co-construction Collaborative Innovation Centre for Chinese Medicine and Respiratory Diseases by Henan & Education Ministry of PR China, Henan University of Chinese Medicine, Zhengzhou, Henan, China; 4 China Academy of Chinese Medical Sciences, Graduate School of China Academy of Chinese Medical Sciences, Beijing, China; 5 The First Affiliated Hospital of Henan University of Chinese Medicine, Zhengzhou, Henan, China; 6 Hongqiang Chinese Medicinal Herbs Planting Professional Cooperative, Shangqiu, Henan, China

**Keywords:** *Fallopia multiflora* (Thunb.), light quality, physiological and biochemical indices, genome-wide analysis, gene expression, secondary metabolism, molecular regulation

## Abstract

Light is one of the most pivotal environmental factors in plant life activities and plays a crucial role in regulating the synthesis of plant secondary metabolites. However, there is no report on the response mechanism of *F. multiflora* to different light quality treatments. In this study, different light qualities were applied. Physiological characteristics and secondary metabolites of plants, including leaf area, chlorophyll, proline, physcion, emodi, THSG etc., were measured after 20-day light quality treatment. Subsequently, the *FmARF* gene family was identified and analyzed the expression under the impact of light quality. Results showed that the blue-light treatment group significantly increased the leaf area by 46.19% and chlorophyll content by 7.35%, decreased the plant height by 20.92%, and increased the THSG content in roots by 28.37% and in stems by 27.15%. The yellow-light treatment significantly increased the proline content by 94.47% and the soluble sugar content by 19.03%. The red-light treatment significantly increased the plant height by 83.56%. There are 37 *FmARF* genes in *F. multiflora*, which are classified into four sub-families. Whole-genome duplication and segmental duplication are the predominant expansion modes. The promoters are abundant in light and other response elements, and most genes show tissue-specific expression. Correlation prediction analysis revealed that in the stems, the content of stilbene glycoside was significantly negatively correlated with *FmARF9* and *30*, and free anthraquinone components such as physcion and emodin were significantly negatively correlated with *FmARF2*, *5* etc. In the roots, the content of stilbene glycoside was significantly negatively correlated with *FmARF8*, 9, 29, and positively correlated with *FmARF17*; physcion was negatively correlated with *FmARF2, 5* etc., and positively correlated with *FmARF8, 9* etc.; emodin was negatively correlated with *FmARF2, 5* etc., and positively correlated with *FmARF9, 15* etc. This study demonstrates that there are significant differences in the growth and development of *F. multiflora* under different light quality treatments. The *FmARF* gene family may mediate light quality regulation and metabolic synthesis pathways. This research provides a theoretical basis for the functional identification of key candidate genes for light regulation and the study of the molecular mechanism of light regulation in *F. multiflora*.

## 1 Introduction

*Fallopia multiflora* (Thunb.), a perennial herbaceous plant in the genus *Fallopia* of the family *Polygonaceae*, is medicinally used with its dried tuberous roots and stems. The dried tuberous roots exhibit effects in preventing and treating hyperlipidemia, atherosclerosis, ischemic stroke, neurological disorders, and other diseases (Zhang and Chen, 2018; Gao et al., 2025). *F. multiflora*-related preparations and washing products have a huge sales market and are used in multiple countries and regions for treating allergies and alopecia (Zhang et al., 2009).

Light quality, as a crucial environmental factor, is one of the key environmental signals regulating plant growth, development, and stress resistance. It influences plant morphological and physiological characteristics at the macroscopic level, as well as internal metabolites and enzyme activities at the microscopic level, through light of different wavelengths during plant growth and development (Soltani et al., 2023). Plant responses to external environmental factors are primarily regulated by transcription factors (TFs). As a class of essential regulatory factors universally present in plants, TFs participate in key physiological reactions such as stress regulatory networks and signal transduction pathways during light-quality-mediated plant growth (Niu et al., 2017). Among them, auxin response factors (ARFs) are regarded as core proteins regulating auxin-responsive genes, playing a vital role in multiple stages of plant growth and development (Tiwari et al., 2003; Garcia-Gonzalez et al., 2020). As key factors in the auxin signaling pathway, ARFs specifically bind to auxin response elements, thereby influencing the expression of auxin-responsive genes in plants (Yu et al., 2014). Studies have shown that ARFs have diverse biological functions and are crucial for regulating various aspects of plant growth and development. For example, *Arabidopsis AtARF1* and *AtARF2* regulate leaf and floral organ senescence (Guilfoyle and Hagen, 2007); *AtARF7* and *AtARF19* control lateral root formation (Wilmoth et al., 2005); rice *OsARF7* is involved in regulating root development (Sun et al., 2025); overexpression of *Phyllostachys edulis PeARF41* reduces the contents of hemicellulose, cellulose, and lignin, thereby inhibiting family secondary cell wall formation (Yang et al., 2025). However, research on the growth and development of *F. multiflora* under light quality regulation and the regulatory mechanism of the ARF gene family in *F. multiflora* growth and development has not been reported.

This study firstly explores the growth, development, and physiological-biochemical changes of *F. multiflora* under light quality regulation, and analyzes the content variations of emodin methyl ether, emodin, stilbene glycosides, and other substances. Additionally, bioinformatics methods are employed to screen and identify members of the ARF transcription factor gene family in *F. multiflora*, and to analyze their phylogenetic relationships, gene structures, physicochemical properties, etc. The expression characteristics of *FmARF* genes in roots, stems, and leaves of *F. multiflora* under different light qualities are analyzed by qRT−PCR, providing a theoretical basis for further research on the response mechanisms of *F. multiflora* at both macroscopic and microscopic levels under light quality regulation. Meanwhile, it also offers a theoretical foundation for the functional identification of key candidate genes involved in light regulation and the exploration of molecular mechanisms of light regulation in *F. multiflora*.

## 2 Materials and methods

### 2.1 Sample collection and processing protocols

The *Fallopia multiflora* (Thunb.) used in this experiment was obtained from the Engineering Technology Research Center for Ecological Planting of Authentic Medicinal Materials at Henan University of Chinese Medicine, Henan Province. The tissue-cultured seedlings were inoculated into a medium composed of 1/2MS, 0.1 mg·L^-1^ NAA, 0.1 mg·L^-1^ IAA, 6 g·L^-1^ agar, and 30 g·L^-1^ sucrose. After 15 days of cultivation in a photoperiod incubator maintained at 25 °C, 45% humidity, and a light intensity of 225 μmol·m^-2^·s^-1^, seedlings with 4−5 true leaves and a plant height of 1−2 cm were selected for treatment. Four treatment groups were established, including yellow light (Y: 590 nm, FWHM: 18.2 nm), white light (W: 450 nm (6500K), FWHM: 25.4 nm), blue light (B: 460 nm, FWHM: 19.5 nm), and red light (R: 660 nm, FWHM: 19.2 nm). All groups were subjected to a consistent light intensity of 225 μmol·m^-2^·s^-1^, and each group had 30 replicates. White light was used as the control group to investigate the core driving effects of monochromatic light on plant photosynthesis and photomorphogenesis. On the 20th day, uniform-growth roots, stems, and leaves tissue samples of *F. multiflora* under different light treatments were collected, immediately treated with liquid nitrogen, and then stored in a -80°C refrigerator.

### 2.2 Determination of growth and physiological indicators of *F. multiflora*

After 20 days of different light quality treatments, growth indices including fresh weight, leaf area, plant height, and color difference of leaf adaxial surface were measured. The contents of chlorophyll a (Chl a), chlorophyll b (Chl b), and carotenoids (Car) in *F. multiflora* leaves were determined by 95% ethanol extraction method: the 3rd fully expanded leaf from the top of the plant was selected. The main vein was removed, and the leaves were cut into pieces. A 0.2 g aliquot of the cut leaves was weighed, followed by the addition of 20 mL of 95% ethanol. The mixture was incubated in the dark for 24 h and inverted to mix evenly. Using 95% ethanol as the blank control, the absorbance was measured at wavelengths of 665 nm, 649 nm, and 470 nm.

The soluble sugar content was assayed using the Solarbio Plant Soluble Sugar Content Detection Kit (Product Lot: BC0030-50T/48s), and the proline content was measured with the Solarbio Proline (Pro) Content Detection Kit (Product Lot: BC0290-50T/48S).

Calculation Formula for Chlorophyll Content:


$${\text{C}}_{\text{Chl}\;\text{a}}=13.95\text{O}{\text{D}}_{665}-6.88\text{O}{\text{D}}_{649}$$

$${\text{C}}_{\text{Chl b}}=24.96\text{O}{\text{D}}_{649}-7.32\text{O}{\text{D}}_{665}$$

$${\text{C}}_{\text{Car}}=\left(1000{\text{D}}_{470}-2.05{\text{C}}_{\text{Chl a}}-114.8{\text{C}}_{\text{Chl b}}\right)/245$$

$$\text{Chlorophyll content }\left(\text{mg}\cdot {\text{g}}^{-1}\text{ FW}\right)=\left(\text{C}\times \text{V}\right)/\left(\text{m}\times 1000\right)$$

C_Chl a_, C_Chl b_, and C_Car_ represent the total concentrations of chlorophyll a, chlorophyll b, and carotenoids, respectively, with the unit of mg·L^-1^.

### 2.3 Changes in secondary metabolite contents of *F. multiflora*

#### 2.3.1 Sample preparation

After freeze-drying the stem and root samples of *F. multiflora* to constant weight and grinding them through a No. 3 sieve, 0.5 g of stem and root samples treated with different light qualities were taken, added to 10 ml of methanol-water solution (90:10, v/v), and subjected to ultrasonic treatment (power 300 W, frequency 40 kHz) for 30 min. After the conical flask was cooled, the weight loss was made up, and the solution was filtered to obtain the test sample.

#### 2.3.2 Chromatographic conditions

Analysis was performed using an Agilent 1260 Infinity III(Column Type: Agilent ZORBAX SB-C18 4.6×250mm,5µm). The gradient elution system consisted of phosphoric acid water (0.1:99.9) (A) and acetonitrile (B), and separation was achieved using the following gradient elution program: 0–10 min: 80% A; 11–15 min: 70% A; 16–30 min: 55% A; 31–35 min: 20% A; 36–40 min: 10% A; 41–60 min: 90% A. The injection volume was 10 μL, the flow rate was 1 mL/min, and the system was operated at 30°C. The detection wavelengths were 320 nm and 222 nm.

#### 2.3.3 Investigation of linear relationship

Methanol solutions containing physcion, emodin, and THSG were prepared and diluted to appropriate concentrations to establish standard curves. The standard regression equations for physcion, emodin, and THSG were *Y* = 79540*X*−34094 (*R*²=0.999, Linear Range:1-40µg·ml^-1^), *Y* = 56678*X*−33868 (*R*²=0.9993, Linear Range: 0.5-20µg·ml^-1^), and *Y* = 25739*X*−32767 (*R*²=0.9998, Linear Range: 2.5-200µg·ml^-1^), respectively. The peak areas obtained from the liquid phase of the three were substituted into the standard regression equations to obtain the compound concentrations and calculate the compound contents.

#### 2.3.4 Precision experiment

An aliquot of the same test sample solution was precisely pipetted and injected consecutively for 6 times. The contents of THSG, emodin, and physcion were determined separately. Their relative standard deviations (RSDs) were calculated to be 0.56%, 1.26%, and 1.52%, respectively, indicating good instrument precision.

#### 2.3.5 Reproducibility test

Six parallel aliquots of the test sample solution were prepared from the same batch of powdered medicinal material, following the established test sample solution preparation protocol. The contents of THSG, emodin, and physcion were determined separately, and their relative standard deviations (RSDs) were calculated to be 1.482578%, 2.089089%, and 2.4724%, respectively. These results confirmed good repeatability of the developed method.

#### 2.3.6 Sample stability

An aliquot of the same test sample solution was precisely pipetted, and the contents of THSG, emodin, and physcion were determined at 0, 3, 6, 12, 24, and 48 hours, respectively. Their relative standard deviations (RSDs) were calculated to be 0.92%, 1.98%, and 1.86%, which indicated that the test sample solution exhibited good stability at room temperature within 48 hours.

#### 2.3.7 Spiked recovery test

The herbal powder sample with a known content was accurately weighed and divided into 6 aliquots. A standard substance equivalent to 100% of the sample content was added to each aliquot. The test sample solutions were prepared following the method described in “Section 2.3.1”, and the recovery rates were calculated under the chromatographic conditions specified in “Section 2.3.2”.Results showed that the recovery rates of THSG, emodin, and physcion were 100.00%, 100.02%, and 100.01%, respectively, with relative standard deviations (RSD) of 2.93%, 1.45%, and 1.35%. These results indicate that the method has good accuracy.

### 2.4 Identification and bioinformatics analysis of the *FmARF* family

The whole-genome annotation files of *F. multiflora* (Zhao et al., 2023)were downloaded from the Figshare database (http://dx.doi.org/10.6084/m9.figshare.19720381), and the reference sequences of *Arabidopsis thaliana AtARF* gene family were obtained from the TAIR database (https://www.arabidopsis.org/). TBtools (Chen et al., 2020) was used for bidirectional BLAST between *F. multiflora* and *Arabidopsis thaliana* to preliminarily screen ARF protein sequences in *F. multiflora*; all protein sequences were downloaded from UniPort (https://www.uniprot.org/) as library sequences, and the screened protein sequences were subjected to Blast alignment again to obtain candidate genes. The Protein Paramter Calc tool of TBtools software was used to analyze the basic physicochemical properties of the *FmARF* transcription factor family.

### 2.5 Analysis of conserved motifs, domains, promoter cis-elements, and gene structures of *FmARF* genes

The conserved motifs in *FmARF* proteins were identified using the Simple MEME Wrapper tool in TBtools, with the motif number set to 10. Conserved domains were determined via the Batch CDD-Search tool on NCBI (https://www.ncbi.nlm.nih.gov/).

The 2,000 bp upstream sequences from the start codons of *FmARF* genes were extracted as promoter regions using the Gft/Gff3 Sequences Extract tool in TBtools. These sequences were submitted to the Plantcare database for cis-acting element prediction. The Gene Structure View (Advanced) function in TBtools was employed to visualize the combined data of domains, motifs, gene structures, and promoter elements.

### 2.6 Chromosomal localization and intra/intergroup collinearity analysis of the *FmARF* gene family

The gene density files were obtained using the Gene Density Profile tool in TBtools. The chromosomal location information of *F. multiflora FmARF* gene members was derived from genome annotation data, and their positions on chromosomes were visualized by Gene Location Visualize (Advanced).

Collinearity files between *F. multiflora FmARF* and *Arabidopsis thaliana AtARF* genes were generated using One Step MCScanX-Super Fast. Chromosomal gene density files were acquired via Gene Density Profile, while gene GC-ratio and GC-skew were obtained by Fasta Window stat. All these data were subjected to visual processing in the Advanced Circos tool.

### 2.7 Expression pattern analysis of *FmARF* family members

#### 2.7.1 RNA extraction and cDNA synthesis

According to the prediction results of promoter cis-acting elements, gene sequences with promoters containing more light-responsive cis-acting elements were selected for expression pattern analysis. The total RNA of *F. multiflora* materials treated with different light qualities on day 20 was extracted using a plant polyphenol and polysaccharide total RNA extraction kit. cDNA was obtained by reverse transcription using the HiScript^®^ III RT SuperMix for qPCR (+gDNA wiper) kit.

#### 2.7.2 Quantitative real-time PCR

##### 2.7.2.1 Primer design

Specific primers for 15 *FmARF* genes were designed using the online primer design program of Sangon Biotech (**Table 1**). The reference gene was PP2A, and the primer sequence information is shown in **Table 1**. All primers were synthesized by Shanghai Sangon Biotech Co., Ltd.

**Table 1:** F*. multiflora* ARF gene primer sequence.

Gene name	Forward primer (5’→3’)	Reverse primer (5’→3’)
*FmARF2*	AGCGGCGGGAAGATGGAGTC	GGAGTTCCGAGGAGGTGGTCTC
*FmARF5*	TTGGTGGTGAGAATGGCGACATG	ACAGAACGTGAAGCGAGTGAACC
*FmARF7*	GCCACCTCCTCCAGCCTTCC	GTCGTCTTCCTCCGCCACAAAG
*FmARF8*	ACGGTTCGGCAACTTGAACAGAG	TCGGTGGTGTAGCGGGATGAG
*FmARF9*	CACGGCGAGGCACTGAGAATC	TGAAGCCCAGCAAACATCCAACC
*FmARF13*	GACAACACGACTTCCGACAGGTG	TCAGCAAGCGGAGGAAGAGGAG
*FmARF15*	GTGGCTGATACTGGGGCAAAGG	CAGCAGCAGCAACAGCAACAAC
*FmARF17*	TGGAAGAGGAGGGTCTGGCTTTG	GGCTGAACCCGACACTGATGAAG
*FmARF19*	AGCACTGAATCGCCAGCAAGAAG	GGACCTTTCGCCACGTCTATCG
*FmARF25*	AACCACACGGCTCCCACCAG	GCTTTCCACATCAGGGTCTGCTC
*FmARF26*	AGGAAGAGGAGGGTCTGGCTTTG	GGCTGAACCTGACACTGACGAAG
*FmARF28*	GTGTAGCGGCGGCATGAGAAG	GTTGGCGTCAGGAGGCTTGC
*FmARF29*	CAGTTCTTGAGCAGGCGGAAGG	AGGCAAGCAAACCACAACCAGAG
*FmARF30*	ATTAGTGGCAGCAGCATGAGCAG	GCTTCTCTTGGGAATCCGTCGTG
*FmARF36*	GCACGACGAGGCACAGAGAAG	TCAGAAGGCAAGGGTCCAGAGG
*PP2A*	GGACCAATGTGCGATCTCTTA	GCTGCTATGTCTTGTCCAAATG

##### 2.7.2.2 Reaction system

7 μL ddH^2^O, 10 μL PowerTrack™ SYBR Green Master Mix, 1 μL forward primer, 1 μL reverse primer, and 1 μL cDNA.

##### 2.7.2.3 Reaction program

UDG enzyme activation at 50 °C for 2 min, pre-denaturation at 95 °C for 2 min, followed by 40 cycles of denaturation at 95 °C for 15 s, annealing at 60 °C for 15 s, and extension at 72 °C for 60 s. Each sample was subjected to three biological replicates.

### 2.8 Data processing

Statistical analysis of qRT-PCR data was performed using Microsoft^®^ Excel 2021. The relative expression levels of genes were calculated via the 2^—ΔΔCt^ algorithm. One-way ANOVA and bar chart visualization were conducted using GraphPad Prism 9.5.0. Heatmap generation was executed with TBtools 2.0.

## 3 Results and analysis

### 3.1 Biomass changes of *F. multiflora*

**Figure 1:**
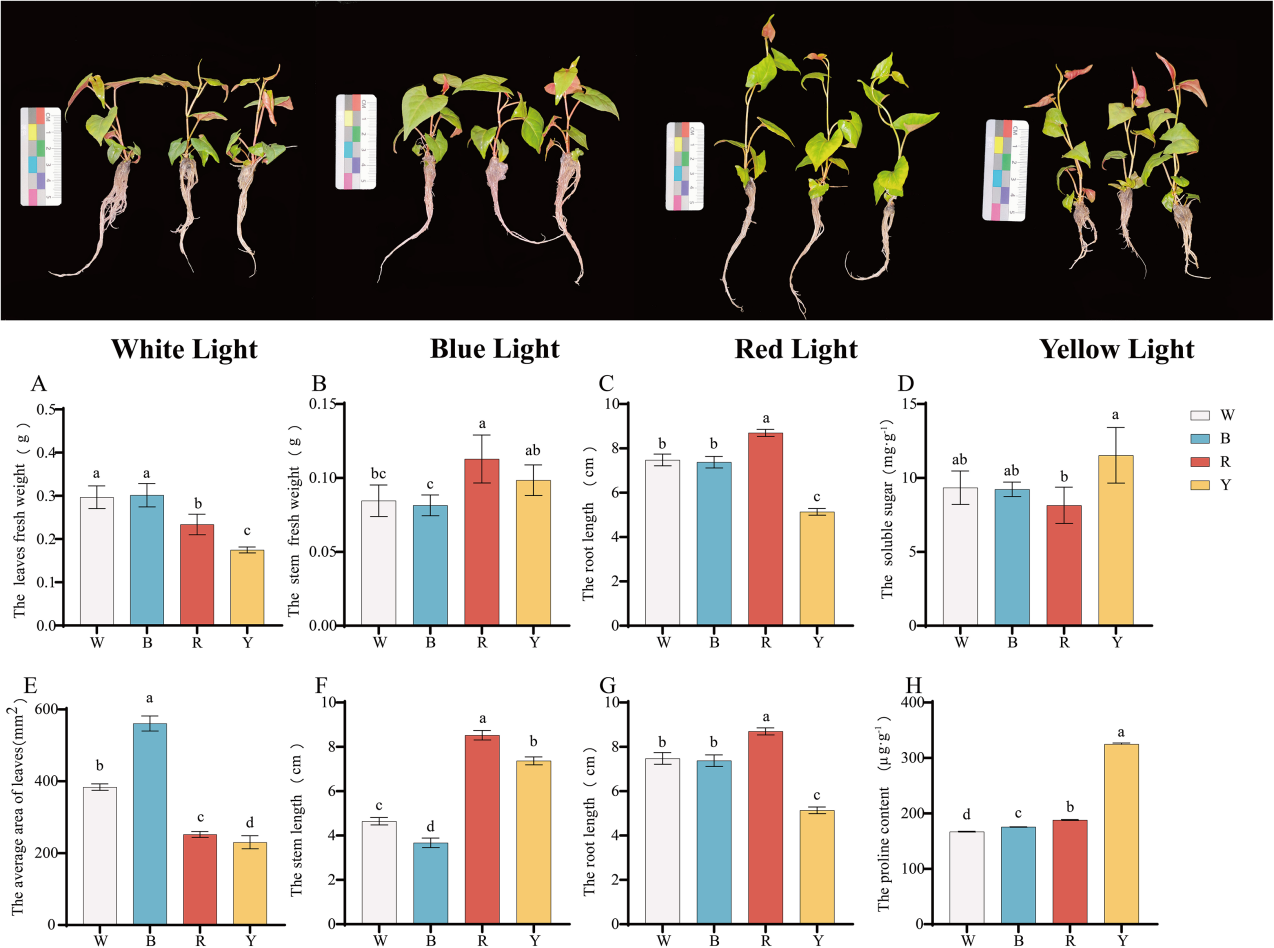
Growth and development changes and osmotic regulator contents in different tissues of *F. multiflora* under the white light (W), red light (R), yellow light (Y), and blue light (B). **(A)** Leaf fresh weight (g). **(B)** Stem fresh weight (g). **(C)** Root fresh weight (g). **(D)** Soluble sugar content (mg-g¹). **(E)** Leaf area (mm²). **(F)** Stem length (cm). **(G)** Root length (cm). **(H)** Proline content (µg-g¹). Values are presented as mean ± standard deviation [SD, n = 5 **(A–C, E–G)** or n = 3 **(D, H)**]. ANOVA results are indicated by a, b, c, d to show significant differences between groups (P < 0.05) using Duncan’s single-factor variance analysis.

The growth and development of *F. multiflora* changed under different light quality treatments (**Figures 1A-C**). Compared with white light, the leaf fresh weight, stem fresh weight, and root fresh weight under yellow light decreased by 41.09%, 16.31%, and 11.85%, respectively; under red light, the leaf fresh weight, stem fresh weight, and root fresh weight decreased by 21.23%, 33.33%, and 34.82%, respectively; there was no significant change in these indicators under blue light compared with white light.

The results indicated that different light quality treatments had a significant effect on regulating the growth and development of *F. multiflora* (P< 0.05). Specifically, red light and yellow light treatments significantly reduced the fresh weight of various tissues in *F. multiflora* (P< 0.05).

### 3.2 Agronomic trait changes of *F. multiflora*

The agronomic traits of *F. multiflora* were determined under different light quality treatments, and significant changes were observed in leaf area and stem length (**Figures 1E, F**, P< 0.05). Blue light treatment significantly promoted the increase in leaf area (P< 0.05), which was 46.19% higher than that under white light treatment. Both red light and yellow light significantly inhibited the increase in leaf area (P< 0.05). Among them, the leaf area under yellow light showed the greatest reduction compared with that under white light, with a decrease of 39.97%.

For stem length, the study indicated that the blue light treatment group had a significant inhibitory effect on stem length (P< 0.05), which was 20.92% lower than that under white light treatment; both yellow light and red light treatments exerted significant promoting effects on stem length (P< 0.05). Among these, the stem length under red light treatment increased the most, with an increase of 83.56%.

Root length was also studied under different light quality treatments (**Figure 1G**). The results showed that red light had a significant promoting effect on root length (P< 0.05), increasing by 16.31% compared with white light; yellow light had a significant inhibitory effect on root length (P< 0.05), decreasing by 31.30% compared with white light; there was no significant difference in root length between blue light and white light treatments.

### 3.3 Chlorophyll and color difference analysis of *F. multiflora*

**Figure 2:**
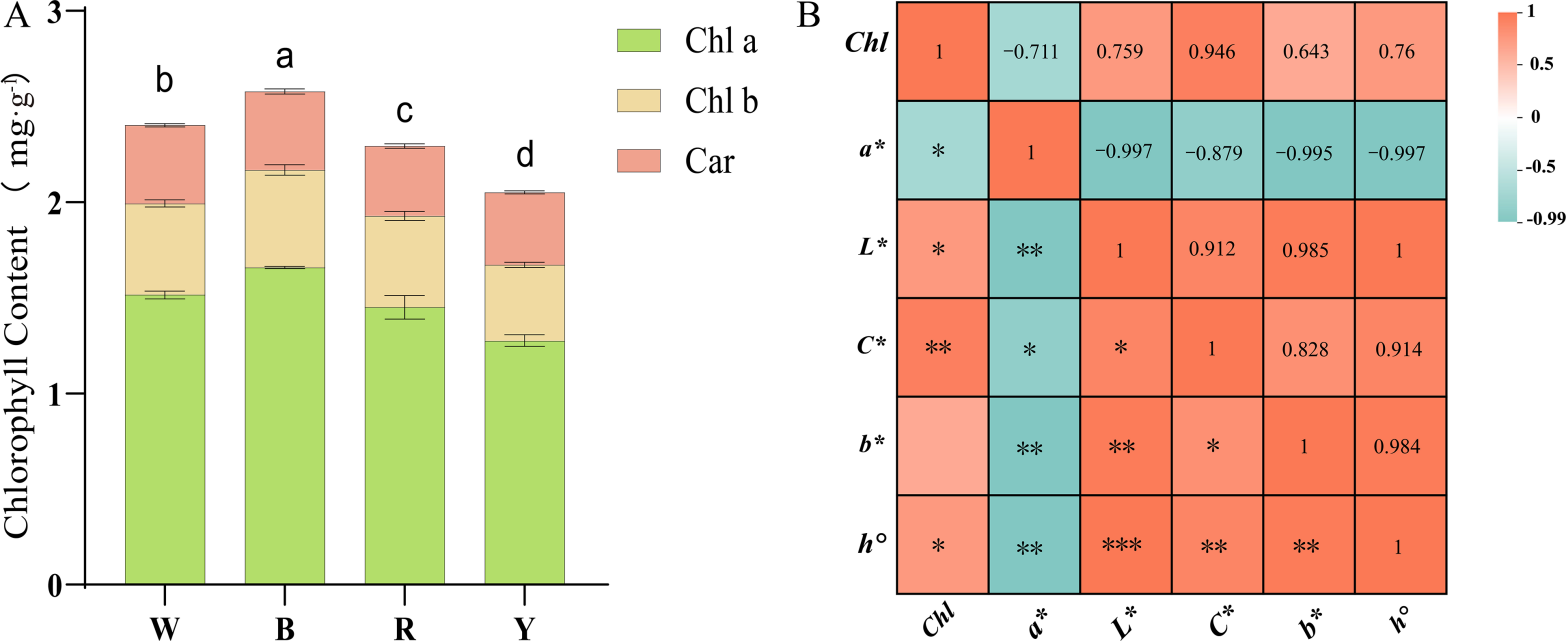
**(A)** Determination of chlorophyll content in *F. multiflora* under the white light (W), red light (R), yellow light (Y), and blue light (B); **(B)** Correlation analysis between chlorophyll content and chromaticity values of the upper surface of *F. multiflora* (The number represents the Pearson coefficient). Data are presented as mean ± standard deviation (SD, n = 3) ANOVA results are indicated by a, b, c, d and **" to show significant differences between groups. (P < 0.05 ; ***p = 0 **p < 0.01 ; *p < 0.05 ) using Duncan's single-factor variance analysis.

The study revealed that light quality had a significant effect on the total chlorophyll content (**Figure 2A**). The blue light treatment group exhibited the highest chlorophyll content, which was 7.35% higher than that under white light. Chlorophyll content decreased under red light and yellow light treatments, with the yellow light treatment group showing the lowest chlorophyll content-14.62% lower than that under white light. Different light quality treatments mainly exerted a significant effect on chlorophyll a (Chl a) content, while their effects on chlorophyll b (Chl b) and carotenoid (Car) contents were relatively minor. Compared with other light quality treatments, blue light was more conducive to the accumulation of chlorophyll a (Chl a).

Quantitative analysis of color difference using a colorimeter revealed that the color difference parameter a* represents the red-green axis chromaticity (**Table 2**). The a* values on the adaxial leaf surface were all negative, with no significant differences among the four treatment groups. However, the blue light treatment group had a higher absolute a* value, indicating a greater tendency toward green. The color difference parameter b* represents the yellow-blue axis chromaticity. Leaf detection showed that b* values were all positive, indicating that leaves tended toward yellow. Significant differences in b* values existed among the four treatment groups. The yellow light treatment group had the largest positive absolute b* value, indicating a stronger tendency toward yellow, while the blue light treatment group had the smallest absolute b* value. The color difference parameter L* represents lightness. The yellow light treatment group had the highest L* value, indicating the highest brightness, whereas the blue light treatment group had the lowest brightness. The color difference parameter C* represents chroma. The yellow light treatment group had the largest C* value, suggesting that leaves became more vivid and saturated after yellow light treatment. The color difference parameter h° represents hue, where values closer to 180° indicate a closer proximity to green. The study found that the h° value of the blue light treatment group was 120.44, closest to 180, indicating greener leaves, while the h° value of the yellow light treatment group was closer to 90°, indicating yellower leaves.

**Table 2:** Chromaticity values of the upper surface of *F. multiflora* leaves.

Treatment	a*	L*	C*	b*	h°
W	-7.64 ± 1.06222a	36.71 ± 1.24357b	17.48 ± 2.21331bc	15.66 ± 2.45998bc	116.36 ± 5.11876b
B	-8.07 ± 0.48023a	33.69 ± 0.86615c	16.04 ± 1.78467c	13.85 ± 1.82801c	120.44 ± 2.21213a
R	-7.385 ± 1.18904a	37.76 ± 0.40273b	19.43 ± 1.39719ab	17.94 ± 1.30483ab	112.37 ± 3.18774bc
Y	-7.38 ± 0.81468a	40.00 ± 1.57059a	21.14 ± 2.20369a	19.80 ± 2.16948a	110.52 ± 2.0493c

W, White light; B, Blue light; R, Red light; Y, Yellow light. a*, Red-green axis chromaticity; b*, Yellow-blue axis chromaticity; L*, Lightness; C*, Chroma; h°, Hue. Data are presented as mean ± standard deviation (SD, n=6).Results of analysis of variance are indicated by a, b, c, d to show significant differences among groups.

Correlation analysis between chlorophyll and color difference parameters was conducted (**Figure 2B**). The study revealed that the red-green axis chromaticity a* showed a significant negative correlation with all parameters (r<0, p<0.05). The smaller the a* value, the greener the *F. multiflora* leaves and the higher the chlorophyll and other index values. Meanwhile, chlorophyll content was correlated with all color difference parameters. Except for the non-significant correlation with the yellow-blue axis chromaticity b* (r=0.984, p>0.05), significant correlations were observed (r>0, p<0.05), indicating that when plant leaves exhibit vivid and bright green colors, chlorophyll content is the highest.

### 3.4 Analysis of osmotic adjustment substances

After different light quality treatments, the soluble sugar content of the plants changed (**Figure 1D**). The yellow light treatment group had the highest soluble sugar content (P< 0.05), while the red light treatment group had the lowest (P< 0.05). There was no significant difference in soluble sugar content between the blue light and white light treatment groups.

Regarding proline content (**Figure 1H**), the yellow light treatment group showed the highest value (324.8118 ± 2.18405 μg·g^-1^), which was extremely significantly higher than that of other treatment groups (P< 0.01) and 94.47% higher than that under white light. The proline content varied greatly among different treatment groups, indicating that different light quality treatments had a significant effect on the osmotic adjustment substances of *F. multiflora*.

### 3.5 Analysis of secondary metabolite contents

Different light quality treatments had significant effects on the formation and accumulation of secondary metabolites in the stems and roots of *F. multiflora* (**Table 3**). Analysis results of stems showed that blue light was conducive to the accumulation of THSG, emodin, and physcion contents in stems. Their contents were significantly higher than those in other treatment groups (P< 0.05), which were 27.15%, 13.56% and 4.09% higher than those under white light, respectively. Analysis of roots revealed that blue light promoted the accumulation of THSG content in roots, which was 28.38% higher than that under white light. Red light treatment was beneficial to the accumulation of emodin and physcion contents in roots.

**Table 3:** Secondary metabolites of *F. multiflora*.

Parts	Treatment	Physcion (µg·g^-1)^	Emodin (µg·g^-1)^	THSG (µg·g^-1^)
Root	W	27.1642 ± 0.22421b	50.2192 ± 1.0471c	388.5233 ± 36.67211b
	B	25.8750 ± 0.43619c	52.0470 ± 0.59193b	498.7853 ± 4.22313a
	R	32.0561 ± 0.12917a	59.6898 ± 0.17445a	338.5681 ± 6.97062c
	Y	24.6801 ± 0.38911d	44.1586 ± 0.3417d	409.6445 ± 5.15667b
Stem	W	380.5958 ± 3.69549b	508.4531 ± 2.67414b	169.1218 ± 0.41833c
	B	396.1444 ± 0.73338a	577.3950 ± 3.10683a	215.0337 ± 0.82855a
	R	303.3087 ± 0.93042c	440.0403 ± 0.97054c	90.9538 ± 0.36353d
	Y	273.5609 ± 0.56481d	388.2959 ± 1.09209d	196.7201 ± 0.26755b

Data are presented as mean ± standard deviation (SD, n=3). Results of analysis of variance are indicated by lowercase letters (a, b, c, d) to denote significant differences among groups.

The results indicated that the contents of emodin and physcion in both stems and roots under yellow light treatment were significantly lower than those in the white light treatment group (P< 0.05). Additionally, the THSG contents in both stems and roots under red light treatment were significantly lower than those in the white light treatment group (P< 0.05).

### 3.6 Analysis of *FmARF* gene family members and their physicochemical properties

Through bidirectional BLAST analysis between *F. multiflora* and *Arabidopsis thaliana*, a total of 37 *FmARF* genes were identified in the *F. multiflora* genome and named *FmARF1* to *FmARF37*. Prediction of their protein physicochemical properties (**Table 4**) revealed that *FmARF14* encoded the shortest polypeptide chain with 358 amino acids, while *FmARF14* encoded the longest with 1116 amino acids. The relative molecular masses ranged from 40,087.57 to 124,334.31 Da. Isoelectric point (pI) analysis indicated that 10 genes (*FmARF2, 3, 4, 6, 8, 11, 18, 19, 22, 32*) had pI values >7, classifying them as basic proteins, while the remaining genes had pI<7 (acidic proteins). Additionally, all family members exhibited negative grand average of hydropathicity (GRAVY) values, indicating hydrophilic properties. The instability index values for all members exceeded 40, classifying them as unstable proteins.

**Table 4:** Physicochemical properties of *F. multiflora* ARF protein family.

Gene ID	Protein length (aa)	Molecular weight (Da)	Isoelectric point (pI)	Instability index	Aliphatic index	GRAVY
*FmARF1*	625	68378.36	6.62	57.26	66.46	-0.281
*FmARF2*	553	61494.48	7.22	56.13	70.89	-0.516
*FmARF3*	422	46934.28	7.16	48.49	75.73	-0.272
*FmARF4*	637	70698.72	7.27	49.16	76.84	-0.318
*FmARF5*	609	67018.02	6.87	51.89	69.01	-0.352
*FmARF6*	567	63283.86	7.23	48.42	74.57	-0.438
*FmARF7*	637	71228.45	6.28	56.94	76.36	-0.368
*FmARF8*	364	40124.28	8.49	44.43	67.03	-0.376
*FmARF9*	811	89874.09	5.22	51.84	73.05	-0.429
*FmARF10*	861	95671.74	5.38	53.94	67.46	-0.509
*FmARF11*	604	66780.16	8.14	53.43	71.34	-0.451
*FmARF12*	901	99646.61	5.4	57.26	74.53	-0.367
*FmARF13*	913	101570.95	5.76	60.31	74	-0.459
*FmARF14*	1116	124334.31	6.02	64.65	68.42	-0.632
*FmARF15*	990	111047.81	6.77	61.73	69.25	-0.656
*FmARF16*	839	93471.21	5.75	57.98	75.76	-0.377
*FmARF17*	658	73612.96	6.28	53.02	67.52	-0.562
*FmARF18*	660	73592.35	7.26	55.26	71.88	-0.467
*FmARF19*	358	40087.57	9.01	60.56	70.89	-0.409
*FmARF20*	606	67877.64	6.03	55.99	67.71	-0.565
*FmARF21*	617	69121	5.66	49.38	67.91	-0.551
*FmARF22*	722	80183.15	8.39	54.59	61.69	-0.624
*FmARF23*	818	90646.07	6.09	60.72	70.05	-0.514
*FmARF24*	782	87649.71	6.16	59.28	74.18	-0.461
*FmARF25*	778	87381.91	5.82	64.22	72.03	-0.513
*FmARF26*	661	73853.48	6.05	53.08	74.15	-0.402
*FmARF27*	876	97845.9	5.63	63.67	70.22	-0.441
*FmARF28*	636	71304.9	5.99	54.77	76.59	-0.461
*FmARF29*	1018	112990.93	6.24	59.52	73.67	-0.567
*FmARF30*	806	90195.53	6.1	59.4	73.88	-0.492
*FmARF31*	594	66825.93	6.22	46.79	74.18	-0.411
*FmARF32*	648	72441.52	7.67	59.82	70.68	-0.522
*FmARF33*	767	84817.65	6.29	53.21	75.37	-0.454
*FmARF34*	711	80197.36	5.44	47.09	73.32	-0.494
*FmARF35*	753	83295.07	6.23	59.86	73.27	-0.449
*FmARF36*	757	84393.57	6.73	54.24	76.7	-0.427
*FmARF37*	603	68730.48	6.75	49.88	74.01	-0.588

### 3.7 Phylogenetic analysis of the *FmARF* gene family

**Figure 3:**
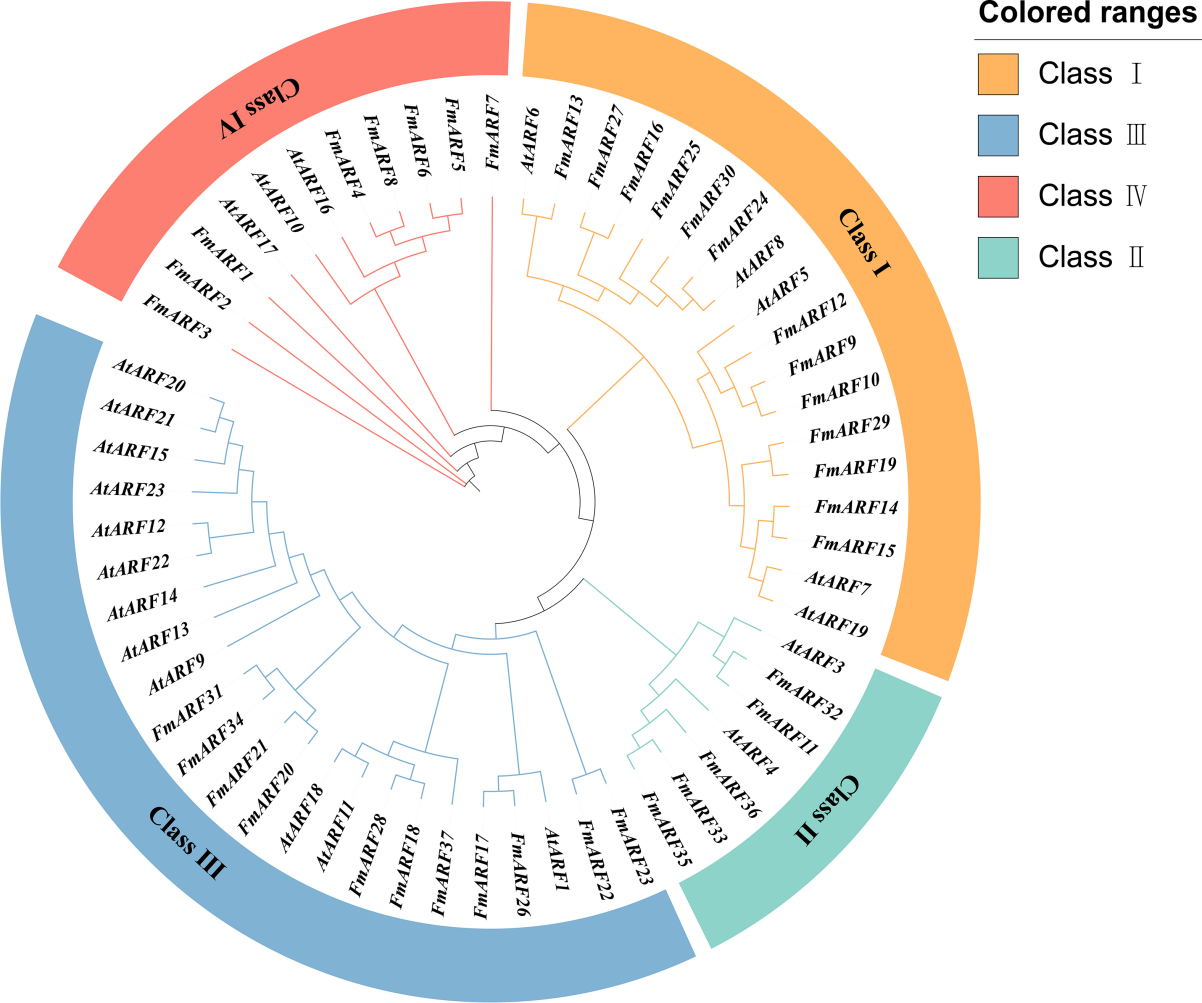
The phylogenetic tree of the ARF (Auxin Response Factor) gene family in F. multiflora. Orange, blue, red and cyan represent Class I, Class II, Class III and Class IV, respectively.

A maximum likelihood (ML) method was used to construct a phylogenetic tree by analyzing the ARF amino acid sequences of *Arabidopsis thaliana* and *F. multiflora* (**Figure 3**). According to the protein structures, the ARF proteins were divided into four subfamilies. The results showed that *FmARF* genes of *F. multiflora* were distributed in all subfamilies, with the largest number of members in Subfamily IV (14 *FmARF* members), followed by Subfamily III (11 *FmARF* members). Subfamily II had the fewest members (5 *FmARF* members), and subfamily I contained 7 *FmARF* members.

### 3.8 Analysis of conserved motifs and gene structures of *FmARF* transcription factors

**Figure 4:**
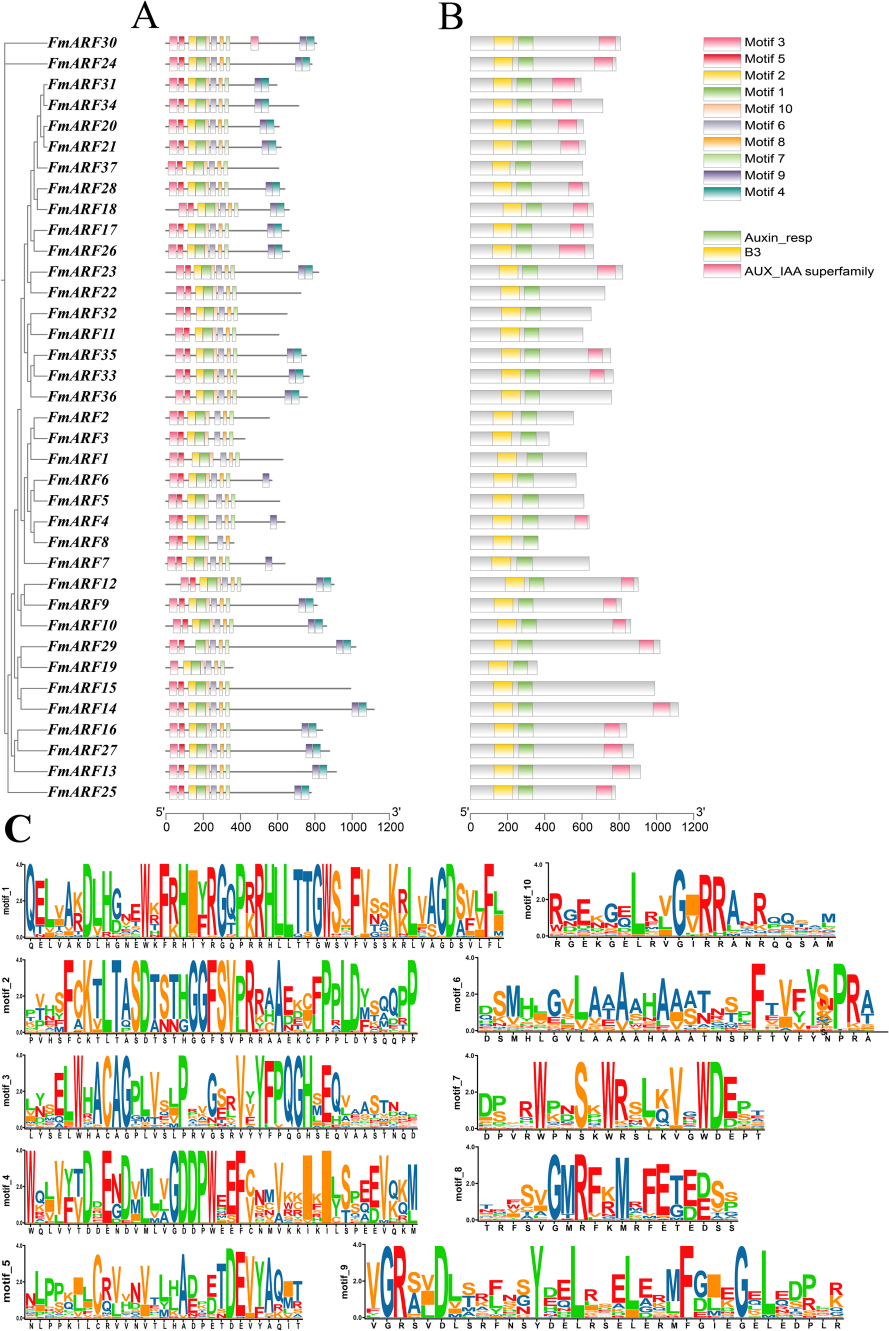
**(A)** Conserved motifs in FmARF proteins; **(B)** Exon-intron structure; **(C)** Amino acid composition of each motif.

Conserved domain analysis revealed that all 37 F*. multiflora* ARF proteins contained both Auxin_resp and B3 domains, indicating these are the most core conserved domains in *FmARF* proteins (**Figure 4B**). AUX_IAA, another critical component, was present in most *FmARF* proteins except for a few members (**Figure 4A**). Using the MEME suite, 10 conserved motifs (Motif 1–10) were predicted across the 37 *FmARF* proteins to dissect their structural diversity and evolutionary relationships. Among these motifs, Motif 1 was the longest, while Motif 8 was the shortest (**Figure 4C**). All *FmARF* proteins harbored Motif 3, 2, 1, 10, 6, 8, and 7, which collectively formed the most highly conserved regions. Specifically, Motif 2, Motif 1, Motif 10, and Motif 6 correspond to the B3 domain; Motif 8 and Motif 7 belong to the Auxin_resp domain; and Motif 9 and Motif 4 are associated with the AUX_IAA domain. Notably, subfamily IV lacked the AUX_IAA domain in all members except *FmARF4*, subfamily II lacked it except for *FmARF35* and *FmARF33*, and subfamilies I and III contained it except for *FmARF22*, *FmARF19*, *FmARF37*, and *FmARF15*. These findings suggest that *FmARF* genes within the same subfamily exhibit structural conservation, whereas distinct subfamilies display pronounced structural divergence.

### 3.9 Prediction of cis-acting elements and analysis of gene structures for *FmARF* transcription factors

**Figure 5:**
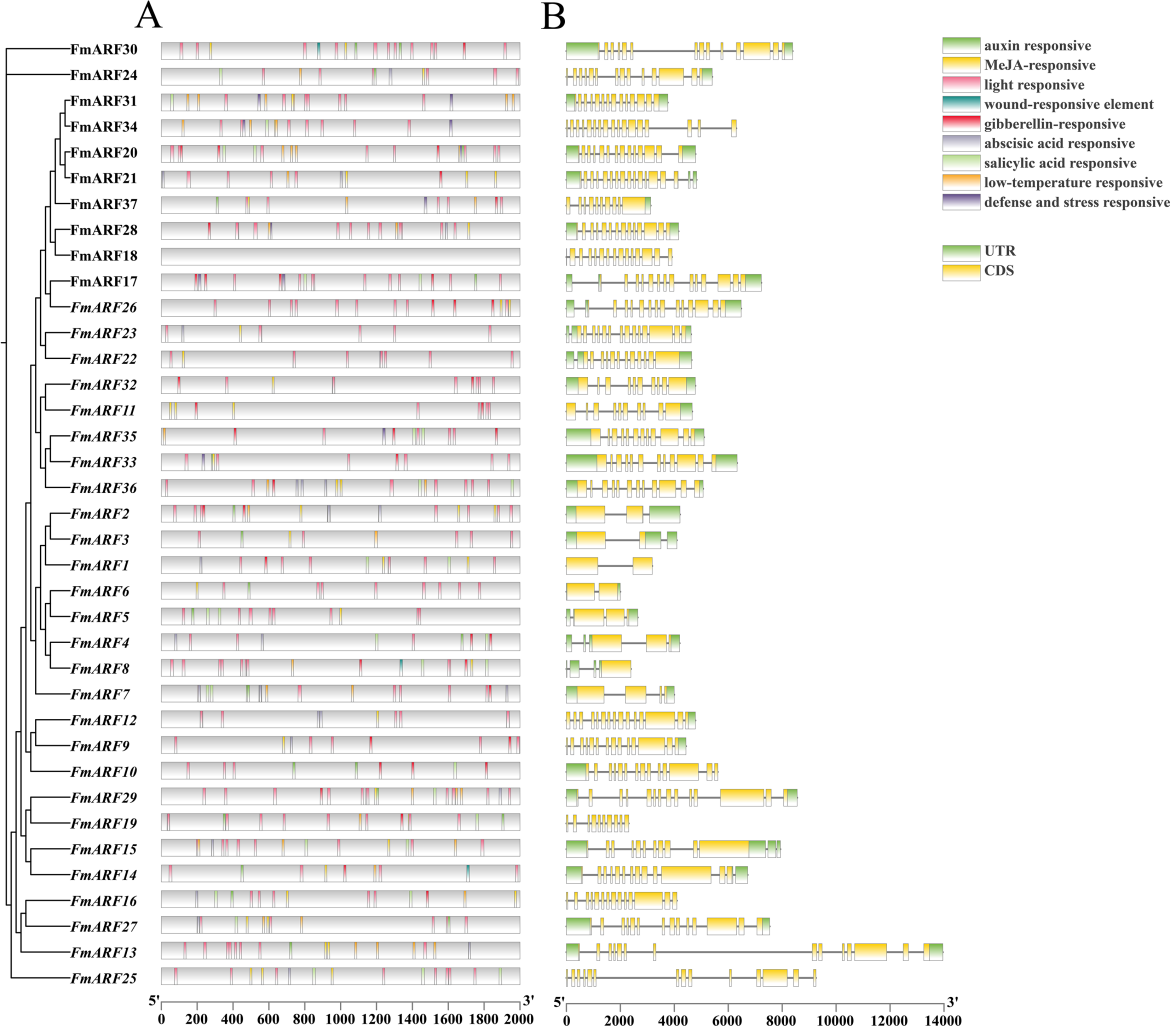
Analysis of cis-acting element of promoter **(A)** and gene structure **(B)** of *F. muliflora* ARF. The different types of cis-acting elements in the promoter region of the FmARFare represented by different colors.UTR and CDS represent untranslated regions and coding sequences, respectively.

The promoter prediction results showed that *FmARF* transcription factors were mainly involved in abiotic stress, hormone, and light regulation responses (**Figure 5A**). A total of 581 cis-elements were identified in all *FmARF* transcription factors, including light-responsive elements, methyl jasmonate-responsive elements, low-temperature responsive elements, etc. Among them, light-responsive elements accounted for the highest proportion at 48.70%, followed by methyl jasmonate-responsive elements at 15.31%. The numbers of the remaining seven types of responsive elements were similar, all accounting for less than 10%. Each gene contained 0 (*FmARF18*) to 26 (*FmARF17*) elements, with the number of types ranging from 0 to 7. Except for *FmARF18*, all *FmARF* transcription factor promoters contained light-responsive elements, among which *FmARF29* and *FmARF28* had a relatively large number of light-responsive elements. The fact that different *FmARF* transcription factors contain different types, numbers, and positions of promoter elements indicates that *FmARF* genes have different functions in plants to complete the process of plant growth and development.

Analysis of the *FmARF* gene structure revealed that the UTR distribution of the *FmARF* gene family was uneven (**Figure 5B**), with the number ranging from 0 (*FmARF18*, *FmARF19*, *FmARF25*, and *FmARF34*) to 4 (*FmARF4*, *FmARF8*, and *FmARF15*). Among them, the number of exons, introns, and CDS of subfamily IV (*FmARF1*-*FmARF7*) was 1-4, which was smaller and more different compared with other subfamilies. This reflects the diversification of genes, indicating that the genes of Subfamily IV are the most primitive ARF genes of *F. multiflora*. With the systematic evolution of *F. multiflora*, the genes have diversified and exercised new protein functions to adapt to new environmental changes.

### 3.10 Chromosomal localization and intra/inter-specific collinearity analysis of *FmARF* genes

**Figure 6:**
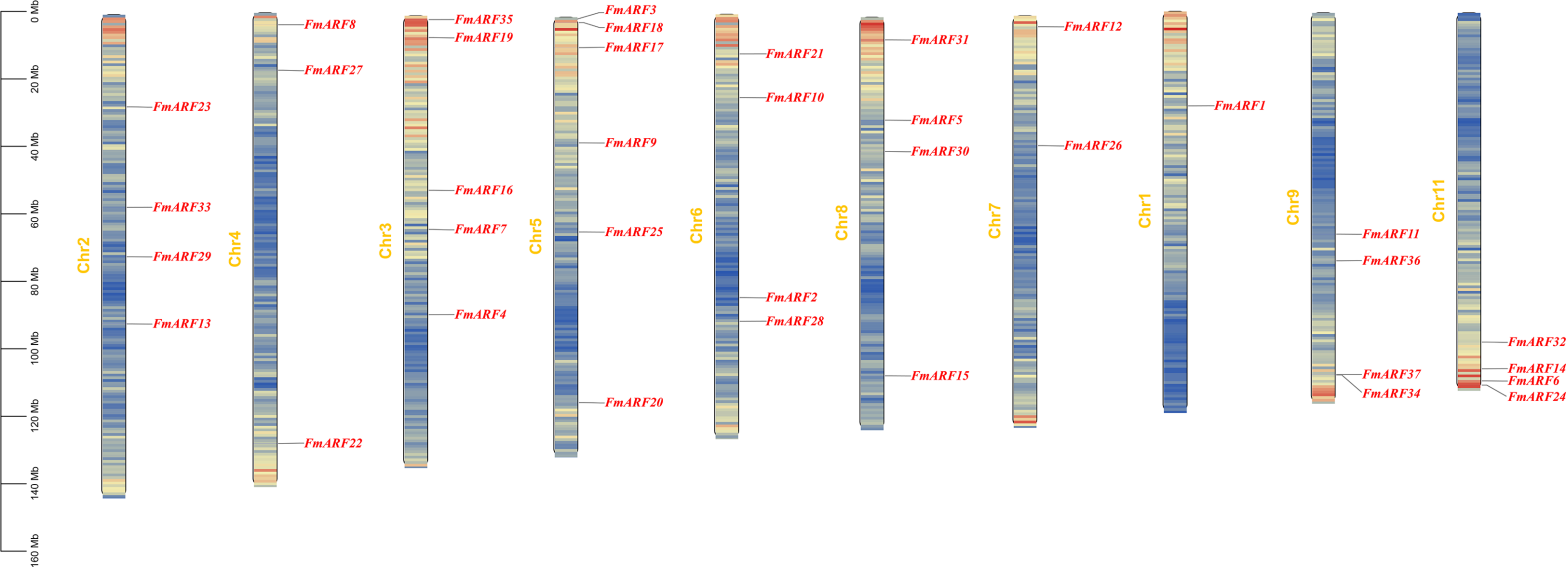
Distribution of *F. multiflora* ARF gene family on chromosomes.

According to the annotation file of *F. multiflora*, the genome assembly of *F. multiflora* consists of 11 chromosomes (**Figure 6**).Thirty-seven *FmARF* genes are unevenly distributed on 10 chromosomes, with the highest number (6 genes) located on Chr5. These results indicate that ARF genes in *F. multiflora* exhibit a dispersed distribution in the chromosome set.

**Figure 7:**
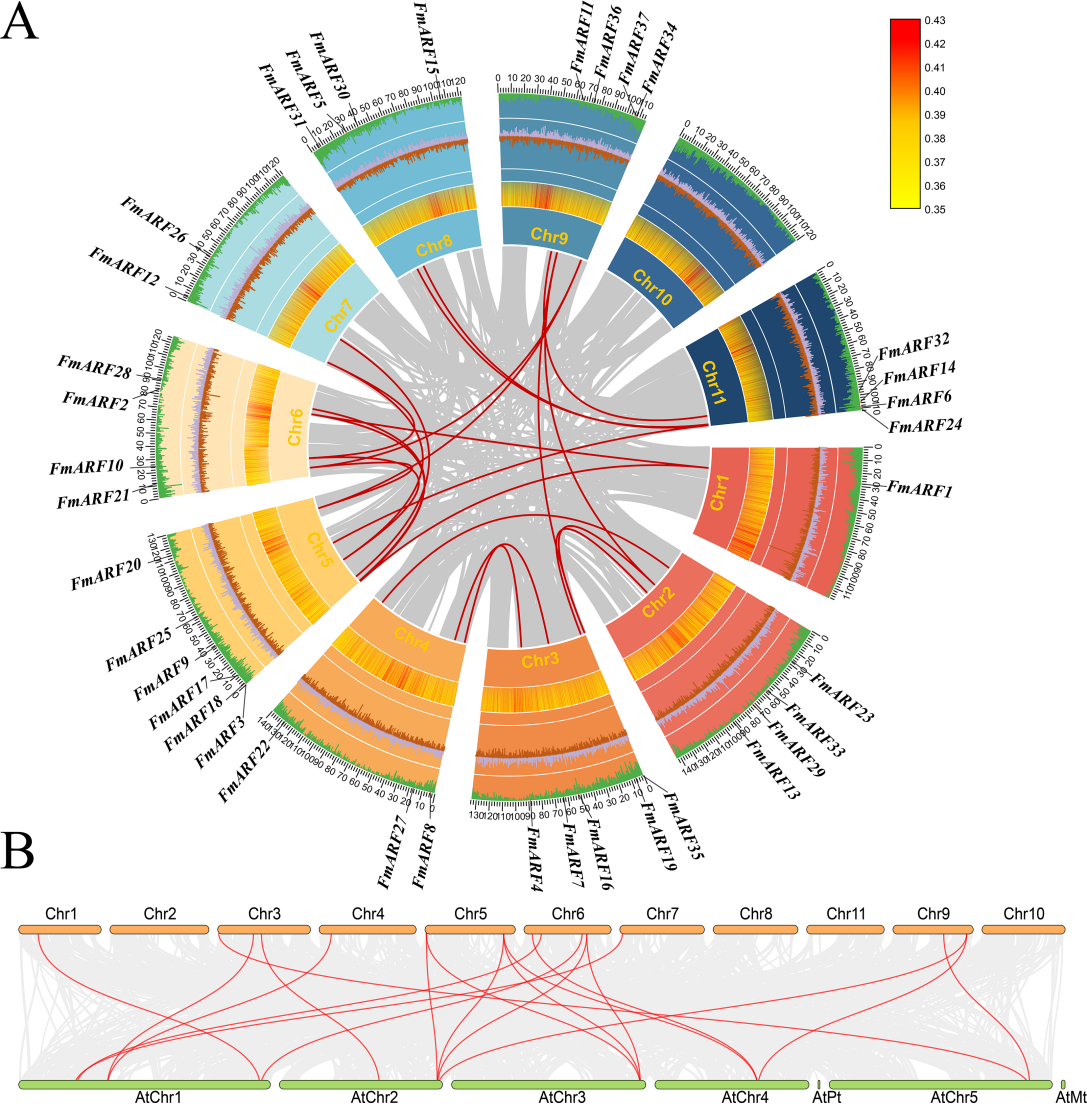
Collinearity analysis. **(A)** Collinearity of the *F. multiflora* ARF gene family in chromosomes, The red line indicates collinearity of FmARF; brown and purple represent GC content offsets, with purple for G > C and brown for G < C; the heatmap shows gene density; cyan denotes GC content proportion; and Chr1-11 are chromosome names; **(B)** Correlation analysis of ARF genes between Arabidopsis thaliana and *F. multiflora*. Gray lines indicate collinearity between the genomes of Arabidopsis and *F. multiflora*, while red lines denote collinearity of ARF genes between the two species.

Collinearity analysis of *FmARF* genes identified 22 pairs of collinear gene relationships (**Table 5**, **Figure 7A**), all resulting from whole-genome duplication and segmental duplication events, with no tandem duplication events detected. Analysis of these 22 FmARF gene pairs showed that the Ka/Ks ratios of all collinear gene pairs were less than 1. This suggests that they have undergone strong purifying selection pressure during evolution, leaning toward purifying selection.

**Table 5:** Collinear relationship among *Fm ARF* genes within species.

Seq 1	Seq 2	Ka	ks	ka/ks	Duplication type
*FmARF37*	*FmARF34*	0.5359	2.2306	0.2403	WGD or segmental duplication
*FmARF1*	*FmARF3*	0.1774	0.6590	0.2692	WGD or segmental duplication
*FmARF1*	*FmARF2*	0.2508	0.7247	0.3460	WGD or segmental duplication
*FmARF3*	*FmARF2*	0.2210	0.6524	0.3387	WGD or segmental duplication
*FmARF6*	*FmARF5*	0.2501	1.3175	0.1898	WGD or segmental duplication
*FmARF9*	*FmARF10*	0.1605	0.8557	0.1875	WGD or segmental duplication
*FmARF9*	*FmARF12*	0.1163	0.6365	0.1827	WGD or segmental duplication
*FmARF10*	*FmARF12*	0.1725	1.0079	0.1711	WGD or segmental duplication
*FmARF32*	*FmARF11*	0.1148	0.5968	0.1923	WGD or segmental duplication
*FmARF16*	*FmARF27*	0.1139	0.5740	0.1984	WGD or segmental duplication
*FmARF17*	*FmARF26*	0.1107	0.5142	0.2153	WGD or segmental duplication
*FmARF18*	*FmARF28*	0.1297	0.6472	0.2003	WGD or segmental duplication
*FmARF20*	*FmARF21*	0.0947	0.5898	0.1606	WGD or segmental duplication
*FmARF20*	*FmARF37*	0.5520	1.7384	0.3175	WGD or segmental duplication
*FmARF21*	*FmARF37*	0.4919	2.1487	0.2289	WGD or segmental duplication
*FmARF23*	*FmARF22*	0.1458	0.5131	0.2842	WGD or segmental duplication
*FmARF24*	*FmARF25*	0.1267	0.8370	0.1514	WGD or segmental duplication
*FmARF24*	*FmARF30*	0.0792	0.7143	0.1109	WGD or segmental duplication
*FmARF29*	*FmARF19*	0.2201	0.8095	0.2718	WGD or segmental duplication
*FmARF33*	*FmARF35*	0.0979	0.5629	0.1740	WGD or segmental duplication
*FmARF33*	*FmARF36*	0.1642	0.7082	0.2319	WGD or segmental duplication
*FmARF35*	*FmARF36*	0.1730	0.6883	0.2514	WGD or segmental duplication

Ka, Non-synonymous substitution rate; Ks, Synonymous substitution rate; ka/ks, The ratio of the non-synonymous substitution rate to the synonymous substitution rate.

Through collinearity analysis between *F. multiflora* and *Arabidopsis thaliana*, the evolutionary relationships of ARF genes between the two species were explored (**Figure 7B**). As shown in the figure, a total of 19 homologous gene pairs were identified. Notably, *FmARF3-6, 8, 9, 11, 13-15, 17, 19, 22-26, 29–34* had no homologous counterparts in *A. thaliana*, indicating that during the evolutionary process, *F. multiflora* has undergone stepwise changes in its ARF gene family to adapt to environmental factors, enabling new protein functions for environmental adaptation.

### 3.11 Expression pattern analysis of light-regulated *FmARF* genes

**Figure 8:**
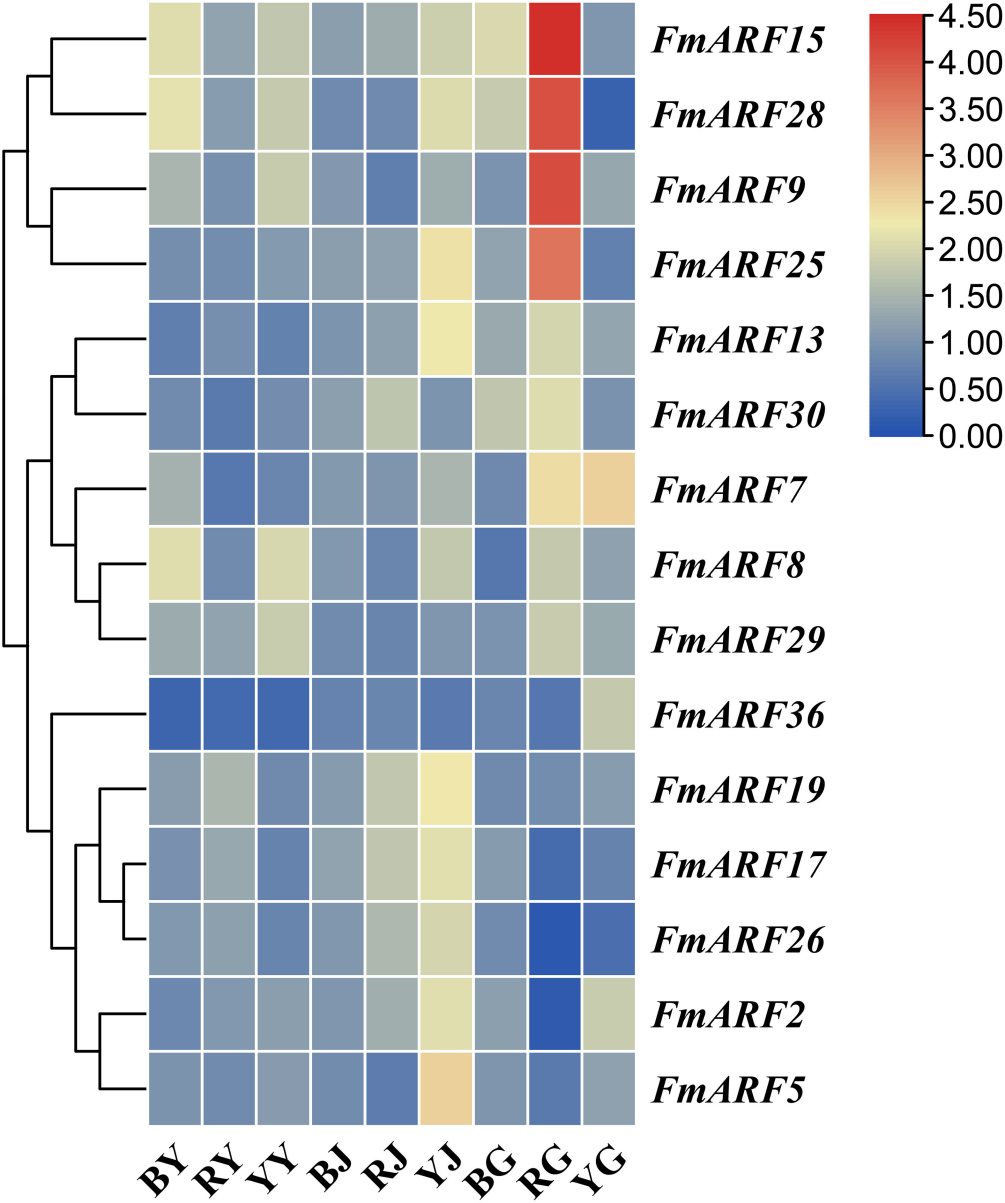
Heatmap of ARF gene family expression levels in *F. multiflora* under different light qualities. (BY: Leaf tissue treated with blue light; RY: Leaf tissue treated with red light; YY: Leaf tissue treated with yellow light; BJ: Stem tissue treated with blue light; RJ: Stem tissue treated with red light; YJ: Stem tissue treated with yellow light; BG: Root tissue treated with blue light; RG: Root tissue treated with red light; YG: Root tissue treated with yellow light.).

Quantitative real-time PCR (qRT-PCR) was employed to determine the expression patterns of 15 genes with abundant light-responsive-responsive cis-acting elements in roots, stems, and leaves of *F. multiflora* under different light quality treatments, using the corresponding tissues from the white light treatment group as controls. Results revealed significant variations in gene expression levels across treatments (**Figure 8**).

Notably, under diverse light qualities: *FmARF36* expression in leaves and stems was significantly reduced compared to white light (P< 0.05); *FmARF13* expression was elevated in roots and stems but decreased in leaves; *FmARF15* expression was universally increased in all three tissues. Additionally, *FmARF28*, *FmARF29*, and other genes were upregulated in leaves; *FmARF2*, *FmARF30*, and others were upregulated in stems; *FmARF9* and other genes were upregulated in roots, while *FmARF26* and others were significantly downregulated in roots (P< 0.05). These findings suggest their potential key roles in mediating light quality regulation in distinct tissues.

Further analysis of the same tissue under varying light qualities demonstrated marked expression differences. For instance: blue light significantly increased *FmARF8* and *FmARF28* expression in leaves (P< 0.05); yellow light led to significantly higher expression of most genes in stems compared to other treatments (P< 0.05); red light induced extremely significant upregulation of *FmARF9*, *FmARF15*, and *FmARF28* in roots (P< 0.01).

In summary, these results indicate that members of this gene family may participate in plant responses to different light wavelengths, regulating tissue development to adapt to light quality changes.

### 3.12 Regulatory network analysis of *FmARF* gene expression and physiological-biochemical indices

**Figure 9:**
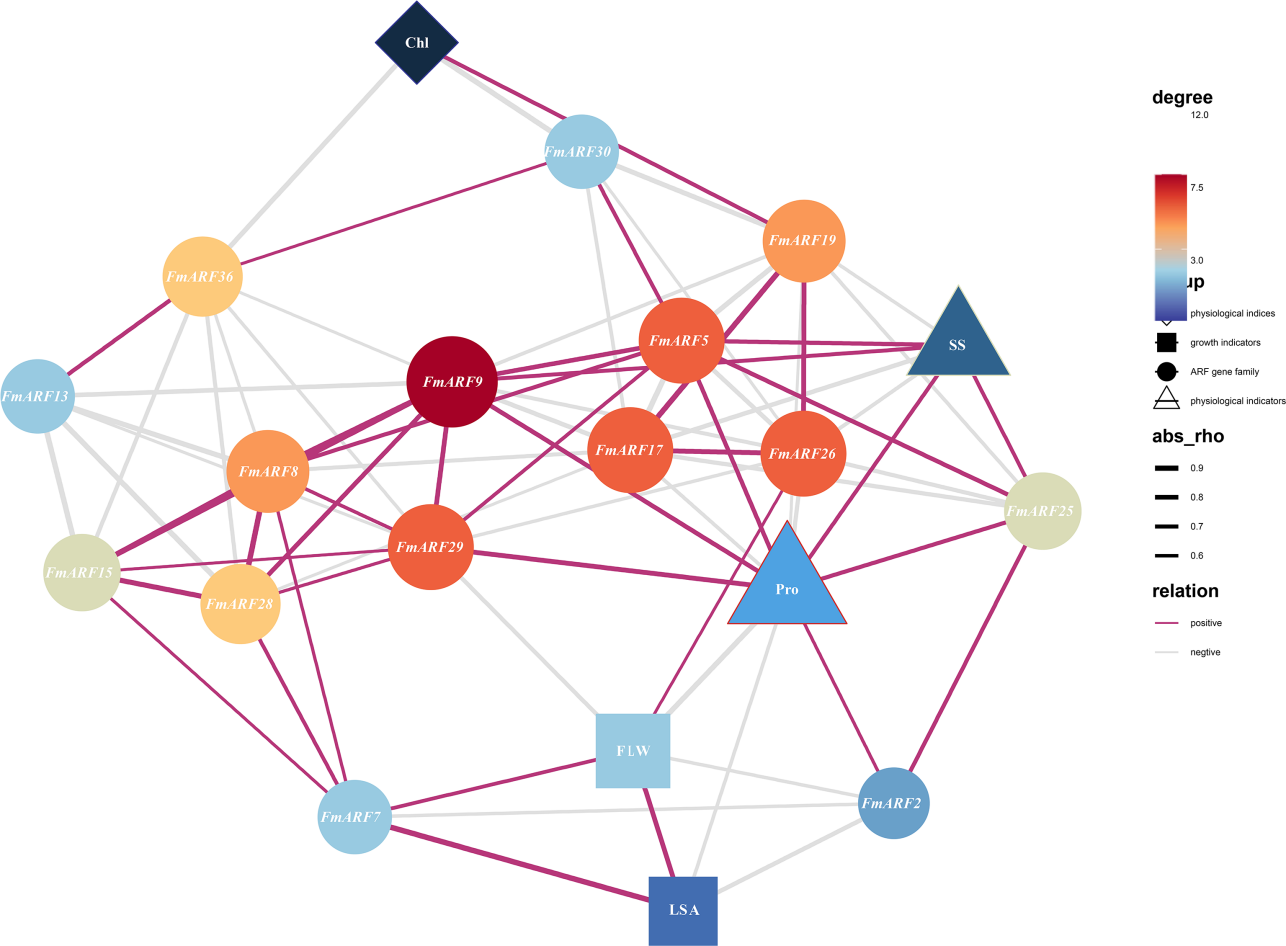
Correlation prediction analysis network heatmap of leaf growth, physiological and biochemical indices, and ARF gene family expression levels in *F. multiflora*. (Chl: Chlorophyll; Pro: Proline; SS: soluble sugar; FLW: Leaf fresh weight; LSA: Leaf area. The color and size of nodes correspond to the degree (connectivity). Nodes that are more orange-red in color and larger in size indicate that the corresponding gene has more associations with other elements).

Correlation prediction analysis was conducted between total chlorophyll content, proline, leaf growth, leaf area, soluble sugar and gene expression indices in *F. multiflora* (**Figure 9**). Results showed that *FmARF9* was correlated with most genes, soluble sugar (r=0.67) and proline (r=0.76) in leaves, and was significantly positively correlated with soluble sugar and proline (P<0.05). Specifically, leaf area (LSA) was extremely significantly negatively correlated with *FmARF2* (r=-0.75, P<0.01) and extremely significantly positively correlated with *FmARF7* (r=0.93, P<0.01). Chlorophyll (Chl) content was significantly positively correlated with *FmARF19* (r=0.68, P<0.05) and extremely significantly negatively correlated with *FmARF36* (r=-0.81, P<0.01). Proline (Pro) was significantly positively correlated with *FmARF2, 5, 9, 25* and *29* (r>0, P<0.05), and significantly negatively correlated with *FmARF17* and 26 (r<0, P<0.05). Soluble sugar (SS) was significantly positively correlated with *FmARF5, 9* and *25*(r>0, P<0.05), and significantly negatively correlated with *FmARF17, 19* and *26* (r<0, P<0.05).

**Figure 10:**
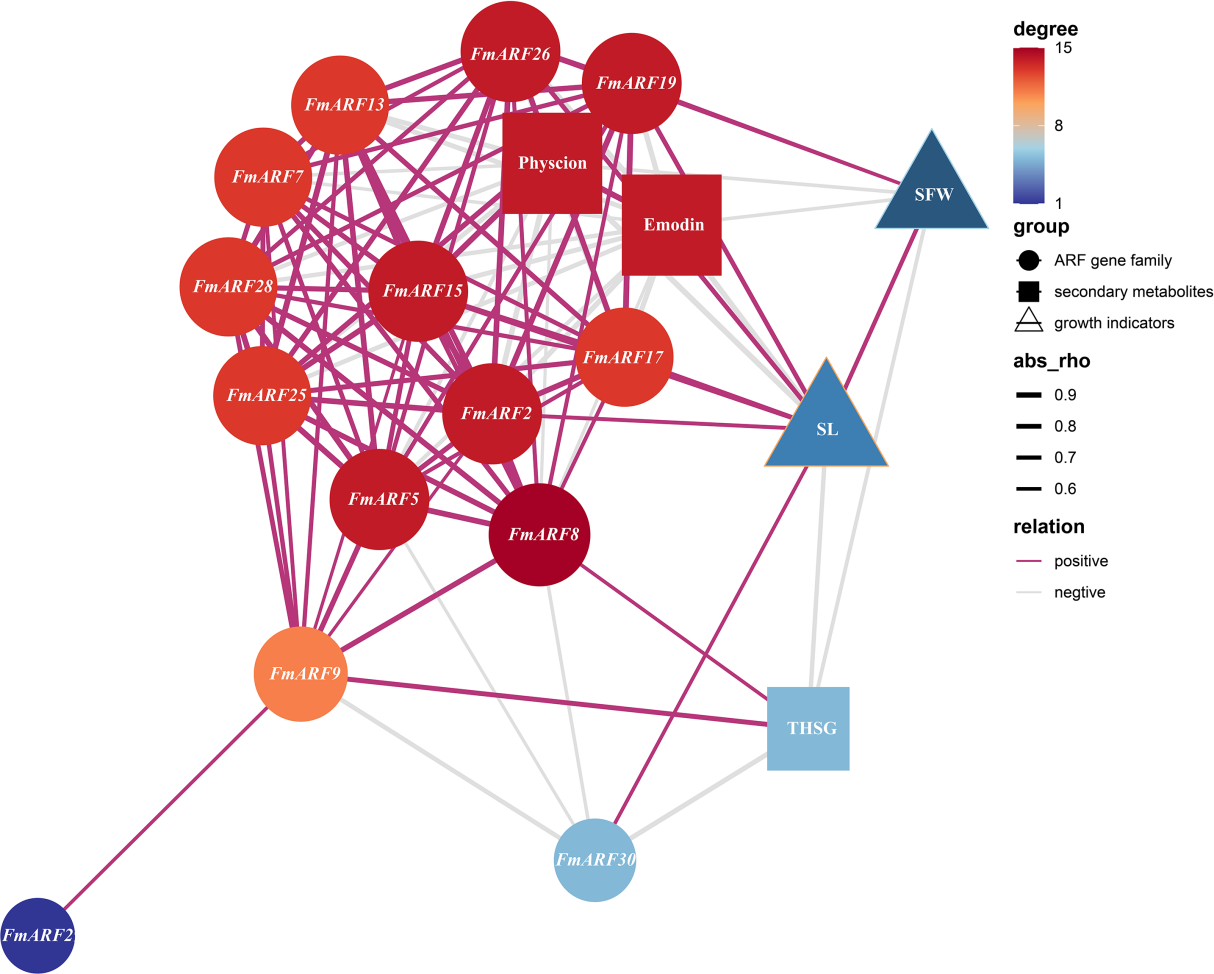
Correlation prediction analysis network heatmap of stem growth, secondary metabolites, and ARF gene family expression levels in *F. multiflora*. (SL: Stem length; SFW: Stem fresh weight. Nodes that are more orange-red in color and larger in size indicate that the corresponding gene has more associations with other elements).

Correlation Prediction Analysis was performed between stem growth (SFW), stem length (SL), physcion, emodin, THSG and gene expression indices in *F. multiflora* (**Figure 10**). Results indicated that *FmARF8* was correlated with most genes, emodin, and THSG in stems. Specifically, stem length was significantly positively correlated with *FmARF17, 19* and *26* (r>0, P<0.05), and significantly negatively correlated with *FmARF2, 15* and *30* (r<0, P<0.05). Physcion showed extremely significant negative correlations with *FmARF2, 13, 15, 17, 19, 25* and *26* (r<0, P<0.01), and significant negative correlations with *FmARF5* and *28* (r<0, P<0.05). Emodin exhibited extremely significant negative correlations with *FmARF2, 13, 15, 17, 19, 25, 26* and *28* (r<0, P<0.01), and a significant negative correlation with *FmARF5* (r<0, P<0.05). THSG showed extremely significant negative correlations with *FmARF30* (r<0, P<0.01) and significant negative correlations with *FmARF9* (r<0,P<0.05).

**Figure 11:**
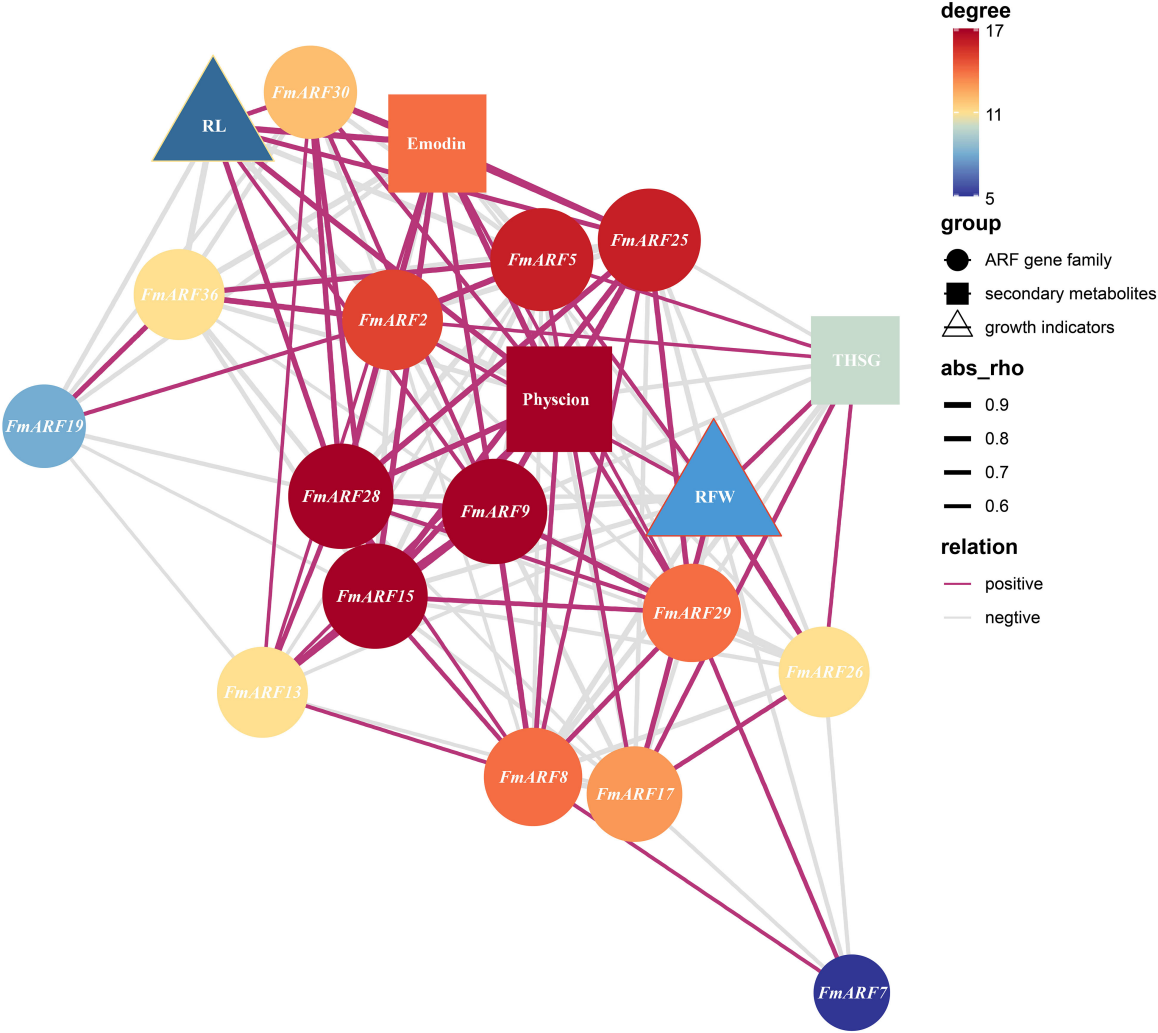
Correlation prediction analysis network heatmap of root growth, secondary metabolites, and ARF gene family expression levels in *F. multiflora*. (RL: Root length; RFW: Root fresh weight. Nodes that are more orange-red in color and larger in size indicate that the corresponding gene has more associations with other elements).

Correlation prediction analysis was conducted between root growth parameters (root fresh weight, RFW; root length, RL) and secondary metabolite contents (physcion, emodin, THSG) against gene expression indices in *F. multiflora* (**Figure 11**). Results showed that FmARF9 was significantly associated with most genes and indices related to emodin and THSG in roots(P<0.05). Specifically, FmARF9 exhibited a significant negative correlation with THSG (r=-0.69, P<0.05) and extremely significant positive correlations with physcion and emodin (r>0, P<0.01).Root length (RL) displayed extremely significant positive correlations with FmARF15, FmARF25, and FmARF28 (r>0, P<0.01), significant positive correlations with FmARF9 and FmARF30 (r>0, P<0.05), and extremely significant negative correlations with FmARF2, FmARF15, FmARF19, and FmARF36 (r<0, P<0.01). Physcion exhibited extremely significant negative correlations with FmARF2, FmARF5, and FmARF36 (r<0, P<0.01), a significant negative correlation with FmARF17 (r=-0.69, P<0.05), extremely significant positive correlations with FmARF8, FmARF9, FmARF15, FmARF25and FmARF28 (r>0, P<0.01), and significant positive correlations with FmARF29 and FmARF30 (r>0, P<0.05). Emodin showed extremely significant negative correlations with FmARF2, FmARF5, and FmARF36 (r<0, P<0.01), a significant negative correlation with FmARF19 (r=-0.67, P<0.05), and extremely significant positive correlations with FmARF9, FmARF15, FmARF25, FmARF28 and FmARF30 (r>0, P<0.01). THSG demonstrated an extremely significant negative correlation with FmARF8 (r=-0.86, P<0.01), significant negative correlations with FmARF9 and FmARF29 (r<0, P<0.05), and an extremely significant positive correlation with FmARF17 (r=0.71, P<0.01).

## 4 Discussion

In the field of plant photomorphogenesis research, *F. multiflora*—a medicinal plant with both shade-tolerant and sun-loving characteristics—has an unclear growth and development mechanism under different light qualities. This study first established white, blue, red, and yellow light treatment systems to systematically analyze the effects of various light qualities on plant morphogenesis, physiological-biochemical indices, and secondary metabolite accumulation in *F. multiflora*. Additionally, ARF gene family members were identified at the whole-genome level, and spatiotemporal expression patterns and functional differentiation of the ARF gene family under different light qualities were deeply explored through physiological index measurements and gene expression analyses. The research predicted the regulatory mechanisms by which *F. multiflora* adapts to light environmental changes from both phenotypic response and molecular regulatory network dimensions, providing critical evidence for improving cultivation ecological regulation strategies of *F. multiflora*. Meanwhile, it illuminates the potential action mechanisms of the ARF gene family in light signal transduction, offering insights for deepening the understanding of plant light signal transduction mechanisms.

As a key driver of plant carbon metabolism, light quality profoundly influences plant morphology, physiology, and various biochemical pathways by regulating light signal transduction (Rehman et al., 2024). Using white light as a reference, this study treated *F. multiflora* with red, blue, and yellow light to systematically analyze the regulatory effects of different light qualities on its growth and development. After 20 days of light treatment, the leaf area of the blue light group significantly increased by 46.19% compared with the white light group (P<0.05), while the leaf areas of the red and yellow light groups were significantly lower than that of the white light group, indicating that different light qualities significantly affect the growth and development of *F. multiflora* leaves. Blue light effectively promotes leaf growth, whereas red and yellow lights inhibit leaf area expansion. In terms of plant height, blue light significantly inhibited vertical growth by 20.92% (P<0.05), while red light significantly promoted plant height by 83.56% and also significantly enhanced root length (P<0.05). Studies on chlorophyll and leaf color difference showed that blue light-treated leaves were greener with the highest chlorophyll content, which was negatively correlated with the a* value, suggesting that blue light promotes chlorophyll synthesis (Liu and van Iersel, 2021). Measurements of osmotic adjustment substances revealed that proline, an important osmotic regulator in plants, accumulated significantly under yellow light to participate in redox reactions, scavenge reactive oxygen species, reduce oxidative damage, and enhance the antioxidant defense system. The change in proline content may be related to the plant light signal transduction pathway. As a specific light quality signal, yellow light may affect the expression of proline synthesis-related genes or metabolic pathways by activating or regulating certain photoreceptors and signal transduction pathways, leading to significant changes in proline content. Additionally, yellow light treatment increased the soluble sugar content in *F. multiflora* leaves, indicating that yellow light promotes plant growth and metabolism, indirectly facilitating soluble sugar accumulation. As an environmental signal, yellow light can be transmitted into cells through photoreceptors, causing changes in gene expression. Some genes related to sugar metabolism may be activated or inhibited under yellow light, affecting the synthesis, conversion, and accumulation of soluble sugars. Systematic studies on active components showed that light quality, as an important environmental regulator, significantly and differentially regulates the accumulation of secondary metabolites in *F. multiflora* (P<0.05). The contents of THSG, emodin, and physcion in stems under blue light were significantly higher than those in other treatment groups. Meanwhile, blue light specifically induced THSG biosynthesis in roots, with its content significantly higher than that in other light quality groups (P<0.05). Previous studies have shown that blue light can affect the expression of flavonoid biosynthesis-related genes in *Epimedium sagittatum* and promote flavonoid metabolite accumulation (Yang et al., 2022). Under different light qualities, significant changes in leaf morphology and chlorophyll content of *Atropa belladonna* occur, and red light significantly promotes the synthesis of tropane alkaloids (Gu et al., 2025). These results reveal the unique roles of different light quality treatments in inducing the accumulation of active components in medicinal plants, providing a theoretical basis for optimizing the light environment regulation strategies for medicinal plants.

Based on the changes in growth and physiological indices of *F. multiflora* under different light qualities, this study further analyzed the auxin response factor (ARF) gene family, a key component of the auxin signaling pathway, to explore its molecular response mechanisms and elaborate on the underlying mechanisms by which it affects plant growth and development at the molecular level. A total of 37 members of the ARF gene family were identified from the whole-genome data of *F. multiflora*. Compared with *Arabidopsis thaliana* (Guilfoyle and Hagen, 2007), rice (Wang et al., 2007), citrus (Li et al., 2015), quinoa (Wang et al., 2025), and tomato (Zouine et al., 2014), the *FmARF* gene family in *F. multiflora* contains a larger number of members. Gene duplication and differentiation are critical for gene family expansion and functional evolution. Collinearity analysis within the genome revealed that the duplication types of the *FmARF* gene family were primarily whole-genome duplication and segmental duplication, with no tandem duplication events detected, consistent with findings in plants such as ginseng (Yan et al., 2023). Analysis of 22 pairs of collinear *FmARF* genes showed that the Ka/Ks ratios of all collinear gene pairs were less than 1, suggesting that they have undergone strong purifying selection pressure during evolution, favoring purifying selection.

Analysis of conserved domains and gene structures revealed that the *FmARF* gene family in *F. multiflora* primarily harbors three major domains: an N-terminal B3-type DNA-binding domain (DBD), which exhibits strong conservation and directly binds to auxin response elements (AuxREs) (Szemenyei et al., 2008); a middle Auxin_resp domain (MR), whose amino acid composition determines whether ARF proteins act as activators or repressors; and a C-terminal dimerization domain (CTD) containing a PB1 (Phox and Bem1) module, which participates in regulating ARF binding to AuxREs and controls the expression of downstream auxin-responsive genes (Tiwari et al., 2003; Roosjen et al., 2018). The ARF gene family achieves precise responses to auxin signals through the collaborative action of these domains: the B3 domain determines the targeting of gene regulation, while the AUX-IAA and Auxin-resp domains control ARF activation timing via dynamic interactions with Aux/IAA proteins. This molecular mechanism enables plants to rapidly adjust processes such as cell growth and organ development in response to changes in auxin concentration.

Promoter prediction of the *FmARF* gene family in *F. multiflora* showed that *FmARF* transcription factors are primarily involved in abiotic stress, hormone, and light regulation responses, indicating that *FmARF* genes participate in related growth and development pathways to regulate the growth and development of *F. multiflora*. As a key factor directly regulating plant growth and development, the regulatory effect of light quality on the ARF gene family has been rarely reported. This study found that under different light quality treatments, the expression levels of ARF genes in different tissues of *F. multiflora* changed significantly. Most ARF genes in the leaves of the blue light treatment group showed significant expression changes, most ARF genes in the stems of the yellow light treatment group were significantly upregulated, and most genes in the roots of the red light treatment group were significantly upregulated. It is speculated that the responses of ARF genes in different tissues of *F. multiflora* vary significantly under different light quality treatments, indicating that these *FmARF* genes play different roles in different tissues under various environmental conditions. Studies have shown that ARF genes in sweet potato (*Ipomoea batatas*) exhibit tissue-specific expression (Pratt and Zhang, 2021),and this result is consistent with the low expression of FmARF8 in the roots of *F. multiflora* observed in this study. Different genes of the same gene family exhibit distinct expression patterns in different tissue parts of plants.

This study conducted correlation analysis between various growth and physiological indices of different tissues and the expression levels of light-regulated ARF genes. The results showed that most ARF genes exhibited negative correlations with the contents of emodin, physcion, THSG and other substances. In the stems of *F. multiflora*, THSG content was significantly negatively correlated with *FmARF9* and *FmARF30*, while free anthraquinones such as physcion and emodin were significantly negatively correlated with *FmARF2*, *FmARF5*, *FmARF13*, *FmARF15*, *FmARF17*, *FmARF19*, *FmARF25*, *FmARF26* and *FmARF28*. In the roots, THSG content was significantly negatively correlated with *FmARF8*, *FmARF9* and *FmARF29*, but positively correlated with *FmARF17*; physcion was negatively correlated with FmARF2, FmARF5, *FmARF17* and *FmARF36*, but positively correlated with *FmARF8*, *FmARF9*, *FmARF15*, *FmARF25*, *FmARF28*, *FmARF29* and *FmARF30*; emodin was negatively correlated with *FmARF2*, *FmARF5*, *FmARF19* and *FmARF36*, but positively correlated with *FmARF9*, *FmARF15*, *FmARF25*, *FmARF28* and *FmARF30*. These strong correlations indicate that the expression of these genes is closely associated with tissue development in different organs and the accumulation of secondary metabolites, suggesting their potential roles in biosynthetic pathways. However, further functional verification is required to confirm whether a causal regulatory relationship exists. However, the correlations between gene expression and secondary metabolite accumulation vary among different tissues, and the mechanisms by which related synthetic pathways respond to these signals and regulate downstream gene expression remain unclear. Further experiments are needed to confirm the functions of these genes and their relationships with response pathways.

In summary, this study explored the growth, development, and physiological changes of *F. multiflora* under different light quality regulations. It was found that growth indices such as leaf area, plant height, and root length of *F. multiflora* showed significant variations under different light quality treatments. The proline content was the highest under yellow light treatment, indicating the strongest stress on *F. multiflora*. Blue light was conducive to the accumulation of THSG in roots and stems of *F. multiflora*, and could promote the accumulation of free anthraquinones such as emodin and physcion in stems.

To analyze the molecular mechanism of growth and development regulation in *F. multiflora*, this study conducted a comprehensive bioinformatics analysis of the ARF gene family in *F. multiflora* at the whole-genome level. A total of 37 *FmARF* genes were identified, which were divided into four subfamilies. The encoded amino acids ranged from 358 to 1116 aa in length, and the relative molecular masses of the proteins were distributed in the range of 40087.57 to 124334.31. All were hydrophilic unstable proteins. All *FmARF* genes contained Auxin_resp and B3 conserved domains. Expression pattern analysis showed that the expression pattern characteristics of *FmARF* genes varied in different environmental conditions and different tissue parts. Correlation analysis between growth, physiological and biochemical indices, and gene expression levels found that the ARF gene family was correlated with the synthesis of secondary metabolites such as physcion, emodin, and THSG in *F. multiflora*. However, further functional verification is required to confirm whether a causal regulatory relationship exists.

This study lays a theoretical foundation for further exploring the regulatory mechanism of different light qualities on the growth and development of *F. multiflora* and the functions of *FmARF* genes in *F. multiflora*.

## Data Availability

The original contributions presented in the study are included in the article/supplementary material. Further inquiries can be directed to the corresponding authors.
